# Knowledge of breast cancer and practice of breast self-examination among adolescent girls in semi-urban area – Savar, Dhaka, Bangladesh: A school-based cross-sectional study

**DOI:** 10.1371/journal.pone.0334375

**Published:** 2026-06-11

**Authors:** Ryadul Alam, Sadia Akter, Tanaa Mohammad, Rowshanara Rini, Mst Mariyam, Afrana Akter Priti, Aishwaria Mitra Ayshee, Md Shahriar Hossen Sayem, Afsana Mohammad Mimi, Ayesha Ahmed, Lakshmi Rani Kundu, Md. Habibullah Talukder, Md. Tajuddin Sikder

**Affiliations:** 1 Department of Public Health & Informatics, Jahangirnagar University, Dhaka, Bangaldesh; 2 National Institute of Cancer Research & Hospital, Dhaka, Bangladesh; Kandahar University, Faculty of Medicine, AFGHANISTAN

## Abstract

Breast cancer is a leading cause of cancer-related deaths worldwide, with most cases in Bangladesh diagnosed at advanced stages due to limited awareness and late detection. While breast self-examination (BSE) is a simple, cost-free method for early detection, its practice among adolescents remains poorly understood. This study aims to explore breast cancer knowledge, BSE practices, and barriers among adolescent girls in a semi-urban area, addressing a critical gap in early prevention efforts. A cross-sectional study was carried out with 384 adolescent girls from four renowned schools in a semi-urban area of Dhaka, Bangladesh. Participants were sampled at school from January to April 2025. Proportionate stratified random sampling was conducted to determine the study sample from each school. A validated semi-structured self-reported questionnaire was employed to collect data from participants during the survey period. Among the participants, only 45.3% had heard of BSE and just 4.6% reported ever practicing it. Overall, breast cancer knowledge was low, with a mean score of 9.49 out of 31 (30.6%). Regression analyses indicated that higher breast cancer knowledge scores were significantly associated with having a science academic background (β = 1.82, p < 0.01), higher family income (β = 1.14, p < 0.05), and prior BSE practice (β = 2.36, p < 0.001). A strong positive correlation was observed between overall knowledge and BSE practice (r = 0.54, p = 0.001). Students from the science section were significantly more likely to practice BSE than those from the humanities section (OR = 9.47, 95% CI: 1.21–73.89, p = 0.032). Participants who received BSE information from health workers were approximately nine times more likely to practice BSE than those who did not (OR = 9.62, 95% CI: 2.30–40.20, p = 0.002). The study demonstrates critically low levels of breast cancer knowledge and BSE practice among adolescent girls, with knowledge strongly influencing practice. These findings underscore the need for culturally appropriate, school-based educational interventions to improve awareness, promote early detection behaviors, and potentially reduce future breast cancer morbidity and mortality in Bangladesh.

## Introduction

Cancer is a major public health problem globally, with an estimated 20 million new cases and 9.7 million deaths in 2022 [1]. Whereas lung cancer is the most common cancer in men (12.4% of all new cases), breast cancer is by far the most common cancer among women, accounting for 11.6% of all new cancer cases [1]. More than half of the breast cancer deaths occur in the economically developing countries [2]. The South Asian countries are experiencing an epidemic of breast cancer that is largely unnoticed. The rate of breast cancer is on the rise and is alarming. About 588 million women aged 15 years or above are affected by a growing breast cancer epidemic. In South Asia, information on epidemiology, genetic and other environmental backgrounds of breast cancer is very limited. Breast cancer in Pakistan affects one out of every nine women, and the mortality rate of breast cancer is 26.0% [3,4]. In India, the percentage of breast cancer incidence is 27% and the mortality is 21.5% [5].

In Bangladesh, breast cancer occupies the 2^nd^ position after cervical cancer and together breast and cervical cancers constitute 38% of all cancers among women. The incidence rate of breast cancer in Bangladesh was 22.5 per 100,000 women [6]. The mean age of patients with breast cancer is 41.8 years, among which reproductive-age women account for 56% [7]. This represents a higher proportion of premenopausal cases in Bangladeshi patients with breast cancer. Even though breast cancer is a leading cause of cancer death among Bangladeshi women, numerous women are largely uninformed of their ailment since social stigma related to female reproductive organs [8]. Around 90% of breast cancers are diagnosed at stage III–IV [9]. This rate of diagnosis at the late stage is concerning and is increasingly becoming common. This may be because of the extreme lack of knowledge, absence of health insurance coverage and ignorance of breast self-screening practices to identify breast cancer at home [4]. Among Bangladeshi women, knowledge about breast cancer and its screening method is low (3.43 ± 2.25 out of a total score of 8), approximately 50% were knowledgeable about the risk factors, only 32.2% of respondents knew at least one breast cancer screening method and 14.7% of women knew about BSE and performed BSE on a regular basis [10]. Sufficient levels of knowledge and awareness of the signs and symptoms and early breast cancer detection, whether by BSE or clinical breast examination (CBE) or mammography are vital in preventing the morbidity and mortality associated with breast cancer [11,12].

In Bangladesh, breast cancer screening has been implemented through clinical breast examination (CBE), a low-cost and easily practicable technique that can be readily taught to healthcare providers in low-resource settings. Presently, 271 Upazila Health Complexes (UHCs) have already introduced the CBE services in all districts in Bangladesh, and the ongoing training is enabling senior staff nurses of 14 other UHCs in 7 other districts to conduct such screening [13]. Mammography and MRI screening are available at some tertiary-level hospitals. However, mammography is expensive and more sensitive for detecting breast cancer among older women [14]. On the other hand, BSE is an easy, expedient, non-invasive and cost-free way for women to detect breast changes [15]. It can be performed on a regular basis, at any age, and is suitable for low-resource countries like Bangladesh, to find any changes in their breasts [16].

The Bangladeshi report of the National Cancer Control Strategy and Plan of Action 2009−15 also supports the findings of the promotion of clinical breast examination (CBE) and BSE as an early detection method of breast cancer, to downstage and improve survival. In addition to that, the Breast Health Global Initiative (BHGI) guidelines for low and middle-income countries recommend BSE as the initial measure in reducing breast cancer risk. Unfortunately, only a few women practice those methods to examine their breasts [17]. Although BSE alone is not an effective tool for early detection of breast cancer, it is simple, non-invasive, convenient, inexpensive and available to all women to detect abnormal lump or mass [11]. However, many women do not even know how to perform BSE [17,18].

Following the current trend, breast cancer accounts for 45% of all cancer among females in the United Kingdom [19]. The incident in the age group between 15–24 is 3.1 per million population in UK [20]. As a rule, in comparison with older women and women above 50, this illness is more common among younger women below 50. The Asian women develop breast cancer at a lower age (40–49 years old), in comparison with Western women aged 50–59 years [21].

Adolescent females are an important target group for the promotion of healthy breast practices, since knowledge and self-care behaviors can be formed during adolescence. However, available studies in Bangladesh mainly focus on adult women and female university students, with limited evidence on adolescents, particularly high-school students in semi-urban settings. Moreover, data on adolescents BSE knowledge, practice, and perceived barriers remain scarce. Therefore, the present study aims to examine the level of knowledge regarding breast cancer and the practice of BSE among female high-school students in a semi-urban area of Bangladesh.

## Methodology

### Study design

This school-based cross-sectional study was conducted among adolescent girls (aged 12–20 years) in a semi-urban area of Dhaka, Bangladesh. Although the World Health Organization commonly defines adolescents as individuals aged 10–19 years, the present study included participants aged 12–20 years based on the age distribution of female students enrolled in the selected secondary and higher secondary institutions during the study period. The study was carried out over a four-month period, from 10 January 2025–10 May 2025, across four government schools that were selected randomly from Savar Upazila, Dhaka District (semi-urban area). The survey was administered in classroom settings during school hours with the permission of the authorities, and each participant required approximately 15–20 minutes to complete the questionnaire.

### Sample size

The minimum sample size was calculated using Yamane’s simplified formula for finite populations:


$$\begin{array}{c} n=\frac{N}{\text{1}+N(e^{\text{2}})} \end{array}$$

$$\begin{array}{c} \text{n}=\frac{\text{9}\text{4}\text{0}\text{0}}{\text{9400}(\text{0}.\text{05})\text{2}} \end{array}$$

$$\begin{array}{c} =\text{383}.\text{7}\approx \text{384} \end{array}$$

Here, *n* is the required sample size, *N* is the total eligible population (9,400; total number of eligible female students in the 4 selected schools) and *e* is the precision level (0.05). Using this approach, the minimum estimated sample size was **384** participants.

The sample size of 384 was proportionately allocated across the four selected schools using the formula,


$$\begin{array}{c} \text{ni}=\;({\text{N}}_{\text{i}}/\text{ N})\;\times \text{ n} \end{array}$$

Where, N_i_ is the number of eligible students in each school, N is the total eligible population (9,400), and n is the final sample size (384). Accordingly, 114 participants were selected from School A, 98 from School B, 86 from School C, and 86 from School D.

### Sampling technique

Two Stage Sampling procedures were used, with each school considered a stratum. The number of participants selected from each school was proportional to the total number of eligible students enrolled, ensuring proportional representation across the study population.

*Stage 1 (school selection):* Four schools in Savar were selected using simple random selection from the list of eligible schools from the existing government secondary school and college list (excluding only boys’ school or college) [22].

*Stage 2 (student selection):* A proportionate stratified random sampling technique was used, with each school considered a stratum. The number of participants selected from each school was proportional to the total number of eligible students enrolled, ensuring balanced representation across the study population.

Inclusion criteria were: (i) female students (class 9–11) who were enrolled in the selected schools, and (ii) willingness to participate in the survey.

Exclusion criteria included participants who provided incomplete responses.

### Study instrument

A pre-tested, semi-structured, self-administered questionnaire with informed consent, sociodemographic information and queries about knowledge of breast cancer and practice of BSE was developed by a thorough review of the literature [23–25]. To introduce more face validity, the questionnaire was selected by an external reviewer who is an oncologist with a long history of consultations with women in Bangladesh regarding breast cancer prevention, diagnosis, and prognosis. The survey was completed following the inclusion of minor revisions according to the feedback of the participants during pre-test.

**Socio-demographic information:** Socio-demographic information was recorded during the survey including age, study year (class), marital status (unmarried/married), family history of breast cancer (yes/no), and relationship with breast cancer affected patient (mother/sister/cousin/aunt/ grandmother).

**Knowledge of breast cancer measures:** To assess the participants’ knowledge of breast cancer, a total of 41 items regarding breast cancer (i.e., 7 for symptoms, 10 for risk factors, 7 for treatment, 8 for prevention, 4 for screening, and 5 for process of BSE) were asked during the survey. Each question has three possible responses (i.e., yes/no/don’t know). Each knowledge item was coded as 1 for correct and 0 for incorrect/don’t know, producing a total score range of 0–41 where higher scores indicate better knowledge. Internal consistency of the knowledge scale was assessed using Cronbach’s alpha (α = 0.935).

**BSE practices:** A single construct (i.e., *Have you ever self-examined your breast for breast cancer*?) was used to assess the BSE with binary responses (yes = 1/no = 0).

### Data analysis

SPSS version 26 was used to analyze the data. Descriptive statistics including frequencies and percentages were calculated with respect to categorical variables; means and standard deviation were calculated with continuous variables. Certain one-way analyses (i.e., t-tests and one-way ANOVA) were calculated to determine the relationship between independent and dependent variables. Pearson correlation test was also conducted to determine the correlations between two continuous variables. To identify factors independently associated with the outcomes, multiple linear regression was performed with the knowledge score as a continuous dependent variable. For BSE practice (binary outcome: Yes/No), binary logistic regression was conducted to estimate adjusted odds ratios (AOR) and 95% CIs. The critical level (p-value) was established at 0.05.

### Ethical considerations

The study protocol was reviewed and approved by the Biosafety, Biosecurity, and Ethical Clearance Committee, BBEC, JU/M 2025/01(175), Jahangirnagar University, Savar, Dhaka-1342, Bangladesh. Data were collected during school hours in classrooms with the permission of the schools authorities. One day prior to data collection, parent/guardian consent forms were distributed via the class teacher (for minors) for home signature and returned the next day; only students (minors) who returned signed guardian consent were chosen to participate. On the survey day, student assent was obtained prior to questionnaire administration.

## Result

### Participant characteristics

A total of 384 female students within age range 12–20 years participated in the study, with the majority aged 15–17 years (78.1%), followed by 12–14 years (11.7%) and 18–20 years (10.2%) (Table 1). Most participants were unmarried (99.2%) and studied in class nine (42.7%) or class ten (34.9%). Students were nearly evenly distributed across science (40.1%), business studies (30.5%), and humanities (29.4%). A minority of only 8.1% reported a family history of breast cancer, most commonly in an aunt (3.1%) or mother/sister (1.8% each). Less than half (45.3%) had heard about BSE, mainly through the internet (26.6%) or friends (4.9%). Alarmingly only 4.4% practice BSE (Table 1).

**Table 1:** Demographic characteristics of Participants.

Variable	n	%
**Age**		
12-14	45	11.7
15-17	300	78.1
18-20	39	10.2
**Study year**		
Class nine	164	42.7
Class ten	134	34.9
Class eleven	86	22.4
**Section**		
Science	154	40.1
Business Studies	117	30.5
Humanities	113	29.4
**Marital Status**		
Married	7	1.8
Unmarried	377	99.2
**Monthly Family Income**		
20,000–30,000 BDT (Lower class)	136	35.4
31,000–50,000 BDT (Middle class)	134	34.9
More than 50,000 BDT (Upper class)	114	29.7
**Father ‘s Education Level**		
Primary	20	5.2
Secondary	100	26.0
Higher Secondary	150	39.1
Graduate	101	26.3
Illiterate	13	3.4
**Mother’s Education Level**		
Primary	42	10.9
Secondary	130	33.9
Higher Secondary	134	34.9
Graduate	72	18.8
Illiterate	6	1.6
**Religion**		
Islam	373	97.1
Hindu	10	2.6
Others	1	0.3
**Family History of Breast Cancer**		
Yes	31	8.1
No	353	91.9
**Relationship with affected Patient**		
Mother	7	1.8
Sister	7	1.8
Aunt	12	3.1
Grandmother	5	1.3
Not applicable	353	91.9
**Have you heard about BSE**		
Yes	174	45.3
No	210	54.7
**Source of Information**		
Television	23	6.0
Radio	2	0.5
Books	5	1.3
Friends	19	4.9
Internet	102	26.6
Health workers	11	2.9
School/ College	12	3.1
Unknown	210	54.7
**Practice of BSE**		
Yes	17	4.6
No	367	95.4

### Knowledge of symptoms, risk factors, and treatment

The mean knowledge score about breast cancer symptoms was 1.71 (SD = 1.82) out of 7, with an overall correct rate of 24.4%. Knowledge of symptoms was significantly higher among older students (18–20 years) (p = 0.004) The most commonly recognized symptom was “swelling of part of the breast” (38.0%), “Sagging of breast” not being a symptom (29.4%),“nipple discharge other than breast milk” (25.0%).However only 13.0% recognized “color changes of the breast including redness or flaky skin. Knowledge of risk factors was similarly low, with a mean score of 1.79 (SD = 2.06) out of 9, with a correct rate of 19.88%. The most frequently identified risk factors were “cyst in the breast” (29.7%) and “family history/genetic reasons” (27.6%), while knowledge of “obesity” (10.7%) and “regular use of birth control pills” (9.1%) was minimal.

The mean knowledge score of treatment was 1.78 (SD = 1.67) out of 7, with a correct rate of 25.4%. Most knew that “breast cancer is curable if detected at an early stage” (41.9%) and that “chemotherapy is an effective treatment” (41.4%). Misconceptions were also evident, with 16.1% believing in alternative medicine and 11.7% in herbal treatment as cures. By contrast, prevention had the highest mean score (2.59 ± 2.12 out of 7) with a correct rate of 37%. More than half of participants recognized “maintaining ideal body weight” (56.8%) and “healthy food habits” (52.6%) as preventive measures, while 44.5% identified “being physically active. “Screening (0.70 ± 1.03 out of 4) with a correct rate of 17.5%. Clinical breast examination was the most recognized method (31.0%), followed by BSE (15.1%). Mammography (9.1%) and ultrasound (14.8%) were the least known (Table 2).

**Table 2:** Distribution of participants’ knowledge about breast cancer’s symptoms, risk, treatment, prevention, screening, and process of breast self-examination.

Variable	Symptoms			Risk			Treatment			Prevention			Screening			Process of BSE		
	Total score = 7			Total score = 10			Total score = 7			Total score = 8			Total score = 4			Total score = 5		
	Mean (SD)	t/F	P value	Mean (SD)	t/F	P value	Mean (SD)	t/F	P value	Mean (SD)	t/F	P value	Mean (SD)	t/F	P value	Mean (SD)	t/F	P value
**Age**																		
12-14	1.42 (1.57)	5.50	0.004*	1.73 (2.04)	0.68	0.505	1.57 (1.49)	0.59	0.553	2.80 (2.13)	0.39	0.675	0.84 (0.99)	0.58	0.591	0.98 (1.36)	0.23	0.794
15-17	1.63 (1.84)			1.75 (2.06)			1.78 (1.72)			2.54 (2.14)			0.67 (1.03)			0.98 (1.36)		
18-20	2.58 (1.71)			2.15 (2.03)			1.97 (1.44)			2.74 (1.97)			0.74 (1.02)			(1.39)		
**Study year**																		
Class nine	1.46 (1.72)	4.30	0.014*	1.53 (1.87)	2.38	0.94	1.63 (1.62)	1.12	0.328	2.17 (2.02)	5.77	0.003**	0.68 (0.98)	0.04	0.958	0.73 (1.17)	2.82	0.06
Class ten	1.69 (1.74)			1.93 (2.07)			1.91 (1.72)			2.85 (2.19)			0.72 (1.04)			1.10 (1.59)		
Class eleven	2.17 (2.03)			2.05 (2.29)			1.82 (1.66)			2.98 (2.06)			0.70 (1.08)			1.01 (1.37)		
**Section**																		
Science	2.14 (2.00)	8.33	<0.001***	2.30 (2.11)	8.56	<0.001***	2.14 (1.68)	7.37	0.001**	2.83 (2.01)	5.63	0.004**	1.07 (1.12)	19.02	<0.001***	1.16 (1.46)	4.12	0.017*
Business Studies	1.28 (1.54)			1.37 (1.79)			1.38 (1.56)			2.05 (2.10)			0.42 (0.76)			0.81 (1.30)		
Humanities	1.54 (1.70)			1.51 (2.08)			1.68 (1.65)			2.82 (2.19)			0.46 (0.97)			0.71 (1.30)		
**Marital Status**																		
Married	0.71 (1.11)	−1.45	0.146	0.14 (0.37)	−2.14	0.032*	0.42 (0.78)	−2.16	0.031*	0.42 (0.78)	−2.748	0.006**	0.00 (0.00)	−1.82	0.069	0.00 (0.00)	−1.79	0.074
Unmarried	1.72 (1.82)			1.82 (2.06)			1.80 (1.67)			2.63 (2.12)			0.71 (1.03)			0.94 (1.39)		
**Monthly Family Income**																		
20,000–30,000 BDT	1.33 (1.52)	7.21	0.001**	1.37 (1.84)	8.32	<0.001***	1.53 (1.62)	6.70	0.001**	2.36 (2.03)	4.32	0.014*	0.50 (0.93)	6.12	0.002**	0.77 (1.28)	4.64	0.10
31,000–50,000 BDT	1.67 (1.81)			1.68 (1.96)			1.61 (1.65)			2.41 (2.13)			0.67 (1.03)			0.80 (1.28)		
More than 50,000 BDT	2.19 (2.04)			2.40 (2.25)			2.24 (1.64			3.07 (2.13			0.95 (1.08)			1.25 (1.55)		
**Father ‘s Education Level**																		
Primary	0.70 (0.97)	1.79	0.128	1.05 (1.57)	4.54	0.001**	0.95 (1.34)	1.515	0.197	1.25 (2.04)	4.22	0.002**	0.35 (0.93)	1.84	0.119	0.55 (1.23)	1.73	0.141
Secondary	1.68 (1.98)			1.16 (1.70)			1.71 (1.65)			2.23 (2.14)			0.56 (0.85)			0.78 (1.12)		
Higher Secondary	1.76 (1.70)			2.06 (2.24)			1.84 (1.71)			2.92 (2.01)			0.84 (1.13)			0.88 (1.39)		
Graduate	1.86 (1.89)			2.13 (1.98)			1.91 (1.64)			2.63 (2.12)			0.70 (0.99)			1.21 (1.55)		
Illiterate	1.53 (1.85)			1.92 (2.32)			1.69 (1.60)			3.38 (2.29)			0.61 (1.19)			0.92 (1.65)		
**Mother’s Education Level**																		
Primary	1.59 (1.34)	0.30	0.873	1.30 (1.86)	2.07	0.084	1.38 (1.44)	0.80	0.526	2.38 (2.14)	1.87	0.115	0.33 (0.78)	1.92	0.108	0.66 (1.26)	2.08	0.082
Secondary	1.59 (1.34)			1.30 (1.86)			1.38 (1.44)			2.38 (2.14)			0.33 (0.78)			0.66 (1.26)		
Higher Secondary	1.60 (2.02)			1.52 (1.96)			1.76 (1.70)			2.30 (2.10)			0.66 (1.01)			0.73 (1.18)		
Graduate	1.80 (1.68)			2.01 (2.15)			1.82 (1.70)			2.85 (2.05)			0.81 (1.07)			1.02 (1.48)		
Illiterate	1.79 (1.89)			2.13 (2.05)			1.94 (1.64)			2.63 (2.19)			0.77 (1.02)			1.19 (1.45)		
**Religion**																		
Islam	1.70 (1.82)	0.49	0.610	1.76 (2.03)	1.87	0.155	1.77 (1.66)	0.10	0.990	2.59 (2.12)	0.32	0.720	0.71 (1.03)	0.43	0.650	0.92 (1.39)	0.42	0.658
Hindu	1.91 (1.91)			2.91 (2.51)			1.81 (1.81)			2.81 (2.34)			0.51 (0.52)			1.21 (1.22)		
Others	0.00			0.00			2.00			1.00			0.00			0.00		
**Family History of Breast Cancer**																		
Yes	1.90 (1.75)	0.63	0.529	1.67 (1.77)	−0.31	0.753	1.67 (1.59)	−0.34	0.732	2.61 (2.09)	0.05	0.958	0.54 (0.81)	−0.85	0.391	1.00 (1.54)	0.30	0.760
No	1.68 (1.82)			1.79 (2.07)			1.78 (1.67)			2.59 (2.12)			0.71 (1.04)			0.92 (1.37)		
**Practice of BSE**																		
Yes	2.82 (1.77)	2.609	0.009**	3.58 (2.09)	3.75	<0.001***	2.82 (1.81)	2.67	0.008**	4.29 (1.57)	3.42	<0.001***	1.76 (1.09)	4.47	<0.001***	2.47 (1.58)	4.83	<0.001***
No	1.65 (1.80)			1.70 (2.01)			1.72 (1.64)			2.51 (2.11)			0.65 (0.99)			0.85 (1.33)		
**Source of Information**																		
Television	1.26 (1.65)	1.35	0.222	2.00 (2.23)	3.89	<0.001***	1.82 (1.66)	9.75	<0.001***	2.47 (2.25)	5.51	<0.001***	0.82 (1.11)	2.54	0.014**	0.91 (1.27)	2.77	0.008**
Radio	1.50 (0.71)			0.50 (0.71)			0.00 (0.00)			1.00 (1.41)			0.50 (0.71)			0.00 (0.00)		
Books	0.60 (0.89)			0.00 (0.00)			0.00 (0.00)			1.20 (1.78)			0.60 (1.34)			0.00 (0.00)		
Friends	1.78 (1.51)			2.21 (1.84)			2.05 (1.22)			3.89 (1.52)			0.94 (0.70)			1.38 (1.55)		
Internet	1.89 (1.83)			2.44 (2.33)			2.68 (1.92)			3.30 (2.23)			0.99 (1.29)			1.27 (1.62)		
Health workers	1.18 (1.32)			0.63 (1.12)			0.72 (0.90)			1.00 (1.41)			0.09 (0.31)			0.09 (0.30)		
School/ College	0.66 (0.49)			0.50 (1.24)			0.25 (0.45)			1.25 (1.95)			0.50 (1.16)			0.33 (0.65)		
Unknown	1.77 (1.92)			1.60 (1.90)			1.50 (1.43)			2.35 (1.99)			0.57 (0.86)			0.82 (1.28)		

Abbreviations: SD, Standard Deviation; BSE, Breast Self-Examination. Independent sample t-test was used for variables with two categories, and one-way ANOVA (F-test) was used for variables with more than two categories. Statistical significance was considered at p < 0.05. *p < 0.05, **p < 0.01, ***p < 0.001.

### Knowledge of prevention, screening, and BSE procedures

Knowledge about process of BSE had a lower mean score (0.93 ± 1.38 out of 5) with a correct rate of 18.5% were the least understood domains. Only 23.7% knew that breasts should be inspected visually in front of a mirror, and 19.5% knew the correct method of palpation while lying down. Around 15% recognized checking the breasts during bathing or feeling for changes in the armpits.

The maximum total knowledge was 31 with a mean of 9.49 (SD = 7.73). Higher knowledge was registered with science students (M = 11.67, SD = 7.83; p < 0.001) higher study years participants (class eleven, M = 10.76, SD = 8.26) higher-income parents and educated fathers. BSE using was very low (4%, M = 0.04, SD = 0.21) The total knowledge was significantly higher among participants who practiced BSE (M = 12.58, SD = 8.41) than non-practitioners (M = 9.49, SD = 7.73; p < 0.001) a factor that indicates knowledge plays a prominent role in enforceable habits (Table 3).

**Table 3:** Participation Total Knowledge and Practice.

Variable	Knowledge			Practice		
	**Mean (SD)**	**t/F**	**P value**	**Mean (SD)**	**t/F**	**P value**
**Age**						
12-14	9.35 (7.69)	1.13	0.323	0.06 (0.25)	1.18	0.306
15-17	9.28 (7.73)			0.46(0.21)		
18-20	11.25 (7.66)			0.00 (0.00)		
**Study year**						
Class nine	8.23 (7.14)	4.00	0.019*	0.04 (0.21)	0.586	0.557
Class ten	10.21 (7.88)			0.05 (0.22)		
Class eleven	10.76 (8.26)			0.02 (0.15)		
**Section**						
Science	11.67 (7.83)	11.86	<0.001***	0.07 (0.26)	3.92	0.021*
Business Studies	7.33 (7.05)			0.03 (0.18)		
Humanities	8.75 (7.53)			0.008 (0.09)		
**Marital Status**						
Married	1.71 (2.56)	−2.70	0.007**	0.00 (0.00)	0.57	0.567
Unmarried	9.63 (7.71)			0.04 (0.20)		
**Monthly Family Income**						
20,000–30,000 BDT	7.88 (7.48)	10.49	**<0.001**	0.02 (0.14)	1.69	0.184
31,000–50,000 BDT	8.87 (7.51)			0.04 (0.20)		
More than 50,000 BDT	12.13 (7.64)			0.07 (0.25)		
**Father ‘s Education Level**						
Primary	4.85 (7.06)	3.53	0.008**	0.00 (0.00)	0.572	0.683
Secondary	8.12 (7.06)			0.03 (0.17)		
Higher Secondary	10.32 (7.72)			0.04 (0.21)		
Graduate	10.45 (8.06)			0.05 (0.23)		
Illiterate	10.07 (7.96)			0.07 (0.27)		
**Mother’s Education Level**						
Primary	7.66 (7.12)	1.80	0.128	0.02 (0.15)	1.21	0.30
Secondary	8.59 (7.62)			0.02 (0.15)		
Higher Secondary	10.32 (7.80)			0.05 (0.22)		
Graduate	10.48 (7.82)			0.08 (0.27)		
Illiterate	11.16 (9.13)			0.00 (0.00)		
**Religion**						
Islam	9.46 (7.71)	0.57	0.566	0.04 (0.20)	0.39	0.673
Hindu	11.10 (8.71)			0.10 (0.31)		
Others	3.00 (0.00)			0.00 (0.00)		
**Family History of Breast Cancer**						
Yes	9.41 (6.81)	−0.05	0.956	0.06 (0.24)	−0.57	0.569
No	9.49 (7.81)			0.04 (0.20)		
**Source of Information**						
Television	9.30 (8.64)	6.29	<0.001***	0.08 (0.28)	0.58	0.770
Radio	3.50 (3.53)			0.00 (0.00)		
Books	2.40 (2.88)			0.00 (0.00)		
Friends	12.26 (5.23)			0.10 (0.31)		
Internet	12.58 (8.41)			0.03 (0.19)		
Health workers	3.72 (4.02)			0.00 (0.00)		
School/ College	3.50 (3.45)			0.00 (0.00)		
Unknown	8.62 (7.20)			0.04 (0.20)		

Abbreviations: SD, Standard Deviation. Independent sample t-test was used for variables with two categories, and one-way ANOVA (F-test) was used for variables with more than two categories. Statistical significance was considered at p < 0.05. *p < 0.05, **p < 0.01, ***p < 0.001.

### Overall knowledge score and associated factors

The overall mean total knowledge score was 9.49 (SD = 7.73) out of 31 equivalents to an overall correct rate of 30.6%. Participants demonstrated relatively better knowledge of prevention compared to symptoms, risk, or treatment, but very poor understanding of screening and BSE procedures. Knowledge scores were significantly higher among science students, those with higher family income and those who practice BSE. Participants who reported practicing BSE had significantly higher knowledge scores than non-practitioners (12.58 ± 8.41 vs. 9.49 ± 7.73; p < 0.001), indicating a positive relationship between knowledge and preventive behavior (Table 3).

### Practice of breast self-examination

**Fig 1:**
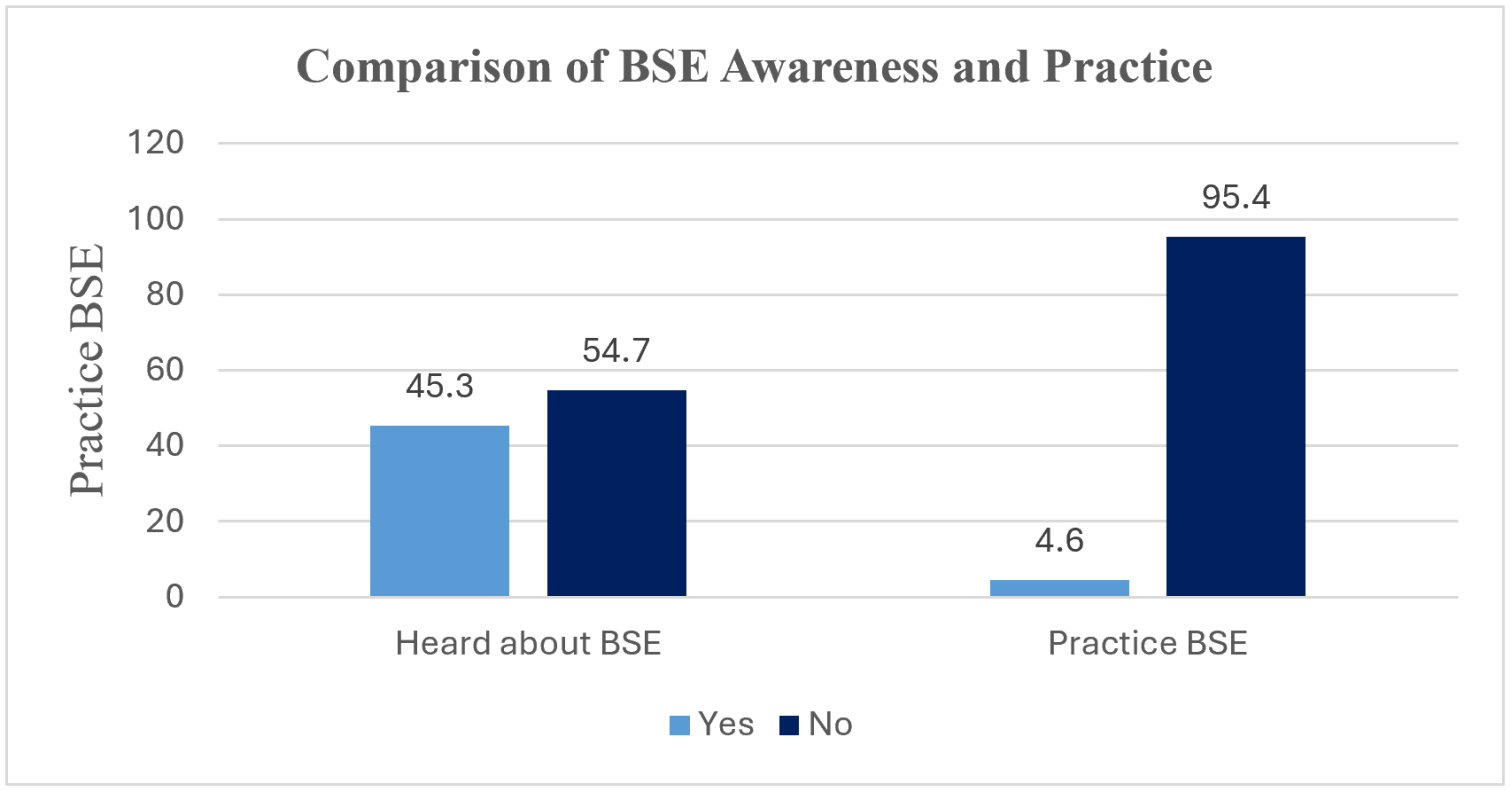
Comparison of BSE Awareness and Practice.

A total of 45.3% of the participants had heard about BSE and 54.7% said they had not heard anything about it. Where only 4.6% practice BSE (Fig 1). There was a significant positive relationship that existed between total knowledge scores and BSE practice (r = 0.54; p = 0.001) which means that the higher the knowledge the better the practice (Table 3).

Descriptive statistics and correlations between all outcome variables (i.e., knowledge about breast cancer’s symptoms, risk, treatment, prevention, screening, and process of breast self-examination, practice of breast self-examination) are presented “Table 4”. Correlation analysis indicated that all knowledge subdomains were significantly correlated with the total knowledge score (r = 0.74–0.82, p < 0.001), suggesting that participants who performed better in one area of breast cancer knowledge also tended to score higher overall. The practice of BSE was weak but significantly correlated with knowledge domains (r = 0.13–0.24, p < 0.001) (Table 4).

**Table 4:** Descriptive statistics, and correlations between all outcome variables.

Variable	M (SD)	Skewness (SE)	Kurtosis (SE)	1	2	3	4	5	6	7	8
Symptoms	1.71 (1.82)	.980 (.125)	–.028 (.248)	–							
Risk	1.79 (2.06)	.926 (.125)	–.151 (.248)	.560**	–						
Treatment	1.78 (1.67)	.620 (.125)	–.576 (.248)	.485**	.614**	–					
Prevention	2.59 (2.12)	.167 (.125)	–1.191 (.248)	.403**	.562**	.609**	–				
Screening	0.70 (1.03)	1.511 (.125)	1.642 (.248)	.482**	.443**	.529**	.455**	–			
Process of BSE	0.93 (1.38)	1.419 (.125)	.939 (.248)	.427**	.462**	.487**	.421**	.454**	–		
Knowledge	9.49 (7.73)	.556 (.125)	–.650 (.248)	.740**	.826**	.818**	.786**	.685**	.684**	–	
Practice	0.04 (0.21)	4.448 (.125)	17.882 (.248)	.132**	.189**	.135**	.173**	.223**	.240**	.231**	–

*SE = standard error; SD = standard deviation.*

** Correlation is significant at the 0.01 level (2-tailed).

### Predictors of total breast cancer knowledge score (multiple linear regression)

Multiple linear regression was conducted to identify socio-demographic and information-related factors associated with total breast cancer knowledge among adolescent schoolgirls. The model was statistically significant overall (F = 5.872, p < .001), explaining 22.5% of the variance in total knowledge (R² = 0.225; adjusted R² = 0.186). In the adjusted model, class of study was positively associated with knowledge (B = 2.252, 95% CI 1.155 to 3.349, p < .001). Monthly family income was also associated with higher level of knowledge (B = 1.636, 95% CI 0.687 to 2.586, p = .001). Heard about BSE showed a significant association with total knowledge (B = −2.601, 95% CI −5.105 to −0.097, p = .042), while as a information sources radio (B = −10.549, 95% CI −20.900 to −0.198, p = .046) and internet (B = −2.573, 95% CI −4.952 to −0.195, p = .034) were also significant predictors. No statistically significant associations were observed for age category, section, parental education, religious affiliation, family history of breast cancer, relationship with the affected patient, television, books, friends, health worker, or school/college status (all p > .05) (Table 5).

**Table 5:** Multiple regression model predicting factors total breast cancer knowledge score among participants.

Predictor	B	Std. Error	β	t	*P*	95% CI for B
Age	−0.023	0.881	−0.001	−0.026	.979	−1.755, 1.709
Study year	2.252	0.558	0.228	4.037	<.001***	1.155, 3.349
Section of class	−0.731	0.479	−0.078	−1.527	.128	−1.673, 0.210
Monthly Family Income	1.636	0.483	0.171	3.390	.001**	0.687, 2.586
Father’s Education Level	0.406	0.490	0.049	0.828	.408	−0.557, 1.369
Mother’s Education Level	0.367	0.475	0.045	0.773	.440	−0.567, 1.302
Religion	3.434	2.129	0.084	1.613	.108	−0.752, 7.620
Family history of breast cancer	−1.266	2.578	−0.045	−0.491	.624	−6.336, 3.804
Relationship with affected patient	0.534	0.972	0.051	0.549	.583	−1.377, 2.444
Heard about BSE (Yes = 1)	−2.601	1.274	−0.154	−2.042	.042*	−5.105, −0.097
Television	0.631	1.654	0.019	0.381	.703	−2.621, 3.883
Radio	−10.549	5.264	−0.098	−2.004	.046*	−20.900, −0.198
Books	−1.424	3.223	−0.021	−0.442	.659	−7.762, 4.915
Friends	−0.415	1.785	−0.012	−0.232	.816	−3.925, 3.095
Internet	−2.573	1.210	−0.147	−2.128	.034*	−4.952, −0.195
Health worker	−0.314	2.302	−0.007	−0.137	.891	−4.841, 4.212
School College	−3.153	1.959	−0.077	−1.609	.108	−7.006, 0.700

Abbreviations: B, unstandardized regression coefficient; β, standardized regression coefficient; CI, Confidence Interval; BSE, Breast Self-Examination. Multiple linear regression analysis was performed to identify predictors of knowledge score. Statistical significance was considered at p < 0.05. *p < 0.05, **p < 0.01, ***p < 0.001.

### Determinants of BSE practice

Academic section showed a significant association with BSE practice. Participants from the science section had significantly higher odds of practicing BSE compared with those from the humanities section (OR = 9.47, 95% CI: 1.21–73.89, p = 0.032). No statistically significant differences were observed for students from the business studies section. Receiving information from health workers was significantly associated with BSE practice. Participants who reported health workers as a source of BSE information had higher odds of practicing BSE compared with those who did not (OR = 9.62, 95% CI: 2.30–40.20, p = 0.002) (Table 6).

**Table 6:** Binary logistic regression analysis of factors associated with BSE practice among adolescent girls.

Predictor	BSE Practice		Χ^2^	*P value*	OR (95% CI)	*P value*
	Yes n (%)	No n (%)				
**Age**						
12–14 years old	3 (6.7%)	42 (93.3%)	2.381	0.304	Reference	
15–17 years old	14 (4.7%)	286 (95.3%)			0.685 (0.189–2.485)	0.565
18–20 years old	0 (0.0%)	39 (100%)			0.00	0.998
**Class of Study**						
Class Nine	8 (4.9%)	156 (95.1%)	1.178	0.555	Reference	
Class Ten	7 (5.2%)	127 (94.8%)			1.075 (0.379–3.044)	0.892
Class Eleven	2 (2.3%)	84 (97.7%)			0.464 (0.096–2.236)	0.339
**Section**						
Science	12 (7.8%)	142 (92.2%)	7.754	0.021*	9.465 (1.212–73.888)	0.032*
Business Study	4 (3.4%)	113 (96.6%)			3.965 (0.436–36.026)	0.221
Humanities	1 (0.9%)	112 (99.1%)			Reference	
**Marital Status**						
Married	0 (0.0%)	7 (100%)	0.330	0.565	–	–
Unmarried	17 (4.5%)	360 (95.5%)			–	–
**Monthly Family Income**						
20,000–30,000 BDT	3 (2.2%)	133 (97.8%)	3.395	0.183	Reference	
31,000–50,000 BDT	6 (4.5%)	128 (95.5%)			2.078 (0.509–8.486)	0.308
More than 50,000 BDT	8 (7.0%)	106 (93.0%)			3.346 (0.866–12.922)	0.080
**Father’s Education Level**						
Primary	0 (0.0%)	20 (100%)	2.303	0.680	–	0.998
Secondary	3 (3.0%)	97 (97.0%)			0.371 (0.036–3.858)	0.407
Higher Secondary	7 (4.7%)	143 (95.3%)			0.587 (0.067–5.178)	0.632
Graduate	6 (5.9%)	95 (94.1%)			0.758 (0.084–6.844)	0.805
Illiterate	1 (7.7%)	12 (92.3%)			Reference	
**Mother Education Level**						
Primary	1 (2.4%)	41 (97.6%)	4.871	0.301	Reference	
Secondary	3(2.3%)	127 (97.7%)			0.969 (0.098–9.568)	0.978
Higher Secondary	7 (5.2%)	127 (94.8%)			2.260 (0.270–18.915)	0.452
Graduate	6 (8.3%)	66 (91.7%)			3.727 (0.433–32.081)	0.231
Illiterate	0 (0.0%)	6 (100%)			–	0.999
**Religion**						
Islam	16 (4.3%)	357 (95.7%)	0.797	0.671	Reference	
Hindu	1 (10.0%)	9 (90.0%)			2.479 (0.296–20.775)	0.403
Others	0 (0.0%)	1(100%)			–	1.00
**Family History of Breast cancer**						
Yes	2 (6.5%)	29 (93.5%)	0.327	0.568	1.554 (0.339–7.129)	0.571
No	15 (4.2%)	338 (95.6%)			Reference	
**Relationship with Patient**						
Mother	0 (0.0%)	7 (100%)	4.080	0.395	–	0.999
Sister	0 (0.0%)	7 (100%)			–	0.999
Aunt	0 (0.0%)	12 (100%)			–	0.99
Grandmother	1 (20.0%)	4 (80.0%)			5.266 (0.556–49.857)	0.148
Unknown	16 (4.5%)	337 (95.5%)			Reference	
**Heard about BSE Practice**						
Yes	8 (7.0%)	107 (93.0%)	2.482	0.115	2.160 (0.812–5.747)	0.123
No	9 (3.3%)	260 (96.7%)			Reference	
**Sources of BSE Information (Multiple Answer)**						
**Television**						
Yes	2 (8.7%)	21 (91.3%)	1.054	0.305	2.197 (0.471–10.245)	0.316
No	15 (4.2%)	346 (95.8%)			Reference	
**Radio**						
Yes	0 (0.0%)	2 (100%)	0.093	0.760	–	0.999
No	17 (4.5%)	365 (95.5%)			Reference	
**Books**						
Yes	1 (20.0%)	4 (80.0%)	2.904	0.088	5.672 (0.599–53.694)	0.130
No	16 (4.2%)	363 (95.8%)			Reference	
**Family/ Friends**						
Yes	0 (0.0%)	19 (100%)	0.926	0.336	–	0.998
No	17 (4.7%)	348 (95.3%)			Reference	
**Internet**						
Yes	5 (4.9%)	97 (95.1%)	0.074	0.786	1.160 (0.398–3.377)	0.786
No	12 (4.3%)	270 (95.7%)			Reference	
**Health Worker**						
Yes	3 (27.3%)	8 (72.7%)	13.969	<0.001***	9.616 (2.301–40.195)	0.002*
No	14 (3.8%)	359 (96.2%)			Reference	
**School/ College**						
Yes	1 (7.1%)	13 (92.9%)	0.253	0.615	1.702 (0.210–13.825)	0.619
No	16 (4.3%)	354 (95.7%)			Reference	

Abbreviations: OR, Odds Ratio; CI, Confidence Interval; BSE, Breast Self-Examination. Chi-square (χ²) test was used to examine the association between categorical variables and BSE practice. Statistical significance was considered at p < 0.05. *p < 0.05, **p < 0.01, ***p < 0.001.

Age group, class of study, marital status, monthly family income, parental education, religion, family history of breast cancer, having heard about BSE, and other sources of information (television, radio, books, internet, family or friends, and school/college) were not significantly associated with BSE practice (p > 0.05) (Table 6).

## Discussion

The present study assessed knowledge of breast cancer and the practice of BSE among adolescent female students in a semi-urban area of Bangladesh. Overall, the findings indicate low levels of breast cancer knowledge across multiple domains and a very low prevalence of BSE practice among the study population. These results highlight important gaps in breast health awareness among adolescent girls in this setting.

Knowledge regarding breast cancer symptoms was limited, although the presence of a breast lump was the most frequently recognized symptom. This finding is consistent with studies conducted in Saudi Arabia, where moderate awareness of breast lump and changes in breast or nipple shape was reported, while other warning signs such as inverted nipple were less commonly identified [26]. Similar patterns have been observed in a school-based study in Sri Lanka, where breast lump was commonly noted as an important warning sign [27]. However, large-scale international studies report substantially higher levels of symptom awareness among women globally [14]. The comparatively lower awareness observed in the present study suggests that symptom-related knowledge among adolescent girls in Bangladesh remains limited relative to other contexts.

Knowledge of breast cancer risk factors was particularly low in this study. This finding aligns with previous research indicating that adolescents often have poor understanding of breast cancer risk factors and limited perception of personal risk [28]. Studies have also shown that although adolescents frequently obtain information from media, peers, and social networks, structured education on breast cancer risk factors is often insufficient [29,30]. In the current study, misconceptions regarding non-evidence-based risk factors were observed, suggesting gaps in accurate health information.

Multiple linear regression analysis identified academic section and monthly family income as independent predictors of breast cancer knowledge scores. Students enrolled in the science section demonstrated significantly higher knowledge scores compared with those in business studies and humanities, even after adjustment for other sociodemographic variables. Additionally, participants from higher-income households had higher knowledge scores than those from lower-income households.

These findings are consistent with studies from Saudi Arabia and other settings, where science students showed better knowledge and attitudes toward breast cancer prevention than non-science students [31]. Socioeconomic differences in cancer awareness have also been reported in low- and middle-income countries, where household income is associated with access to health information and educational opportunities [32,33]. Nevertheless, as knowledge acquisition is influenced by multiple social and contextual factors, these associations should be interpreted cautiously.

A moderate proportion of participants demonstrated awareness of early diagnosis and treatment of breast cancer. Comparable findings have been reported in studies conducted in Iraq, where awareness of conventional treatment options such as chemotherapy, surgery, and hormonal therapy was limited [34]. In contrast, studies among female university students in Egypt reported higher awareness of standard treatment modalities [24], possibly reflecting differences in age, educational level, and exposure to health education.

Notably, a subset of participants in the present study believed that alternative or herbal treatments could be effective for breast cancer. Similar misconceptions have been reported in other low- and middle-income countries and may influence treatment-seeking behavior, although this study did not directly assess health-seeking outcomes or treatment decisions.

Awareness and practice of BSE were low among participants. Although nearly half of the respondents had heard of BSE, only a small proportion reported ever practicing it. This finding is consistent with previous studies in Bangladesh, which reported moderate awareness but low practice of BSE among female students and young women [17,35]. The discrepancy between awareness and practice has been widely documented and may reflect limited confidence, inadequate practical instruction, or perceived lack of relevance among adolescents.

Binary logistic regression analysis revealed that academic section and exposure to health workers were significantly associated with BSE practice. Students from the science section had higher odds of practicing BSE compared with those from the humanities section. In addition, participants who reported receiving information from health workers were significantly more likely to practice BSE than those who did not.

These findings suggest that educational background and access to credible health information sources may influence preventive health behaviors among adolescents. Similar associations between contact with health professionals and increased likelihood of BSE practice have been reported in other studies [33,36]. However, given the cross-sectional design and the small number of participants reporting BSE practice, these associations should be interpreted with caution.

The study also identified a significant association between overall breast cancer knowledge scores and BSE practice. While this association indicates that higher knowledge levels coexist with a greater likelihood of reported BSE practice, the direction of this relationship cannot be determined. Due to the cross-sectional nature of the study, it is not possible to establish whether increased knowledge leads to BSE practice or whether individuals who practice BSE subsequently seek more information.

In Bangladesh, access to population-based breast cancer screening methods such as mammography is limited and not routinely feasible for adolescents. In this context, BSE may serve as an accessible breast awareness tool rather than a diagnostic screening method. However, the effectiveness of BSE as a screening strategy was not evaluated in this study, and its role should be interpreted within the broader framework of breast health education.

### Limitation

In this study, all data were self-reported by the participants, and this brings about the issue of recall bias. There was no independent check to determine the validity of the claims made by participants about the practice of BSE or not. The cross-sectional design precludes causal inference between knowledge and BSE practice. Additionally, as the study was conducted among adolescent girls in semi-urban schools, the findings may not be generalizable to other populations in Bangladesh. It is possible to suggest future research with bigger samples, different populations (e.g., adolescent girls who live in poverty, adolescent girls who represent various socio-economic groups) and longitudinal or mixed-method designs, which could help these limitations and give more information.

## Conclusions

This study shows that adolescent girls living in semi urban areas knew little about breast cancer, between 27.4%, 24.36% and 17.94% regarding treatment options, symptoms and risk factors respectively. The reported practice of BSE was very low (4.4%). A significant association between breast cancer knowledge and BSE practice was observed, suggesting that higher knowledge co-occurs with greater likelihood of practicing BSE, although causal relationships cannot be inferred.

These findings highlight a need for further research to assess baseline knowledge and BSE practices among broader adolescent populations and different socio-demographic groups in Bangladesh. Culturally and educationally appropriate interventions may be considered to improve awareness and understanding of breast cancer and to support informed health practices among adolescent girls.

## Supporting information

S1 File Supplementary tables describe knowledge about breast cancer symptoms, risk factors, treatment, prevention, screening, and breast self-examination among participants. (DOCX)

## References

[1] World Health Organization. WHO: Global cancer burden growing, amidst mounting need for services. https://www.who.int/news/item/01-02-2024-global-cancer-burden-growing--amidst-mounting-need-for-services. 2024. PMC11115397 38438207

[2] Godfrey K, Agatha T, Nankumbi J. Breast cancer knowledge and breast self-examination practices among female university students in Kampala, Uganda: A descriptive study. Oman Med J. 2016;31:129–34. doi: 10.5001/omj.2016.25 27168924 PMC4861385

[3] Naqvi AA, Zehra F, Ahmad R, Ahmad N. Developing a Research Instrument to Document Awareness, Knowledge, and Attitudes Regarding Breast Cancer and Early Detection Techniques for Pakistani Women: The Breast Cancer Inventory (BCI). Diseases. 2016;4(4):37. doi: 10.3390/diseases4040037 28933416 PMC5456323

[4] Naqvi AA, Zehra F, Ahmad R, Ahmad R, Ahmad N, Yazdani N, et al. Awareness, knowledge and attitude towards breast cancer, breast screening and early detection techniques among women in Pakistan. J Pak Med Assoc. 2018;68(4):576–86. 29808048

[5] Jadhav CR, Srinivasamurthy BC, Sb KP, Agrawal V, Kv B. Breast tumors and college students: a study of their knowledge, attitude and practice. Indian J Pathol Oncol. 2017.

[6] Begum SA, Mahmud T, Rahman T, Zannat J, Khatun F, Nahar K, et al. Knowledge, Attitude and Practice of Bangladeshi Women towards Breast Cancer: A Cross Sectional Study. Mymensingh Med J. 2019;28(1):96–104. 30755557

[7] Hossain MS, Ferdous S, Karim-Kos HE. Breast cancer in South Asia: a Bangladeshi perspective. Cancer Epidemiol. 2014;38(5):465–70. doi: 10.1016/j.canep.2014.08.004 25182670

[8] Social taboo leading cause for unchecked breast cancer in Bangladesh. Dhaka Tribune. https://www.dhakatribune.com/bangladesh/event/159421/social-taboo-leading-cause-for-unchecked-breast

[9] Story HL, Love RR, Salim R, Roberto AJ, Krieger JL, Ginsburg OM. Improving outcomes from breast cancer in a low-income country: lessons from bangladesh. Int J Breast Cancer. 2012;2012:423562. doi: 10.1155/2012/423562 22295245 PMC3262600

[10] Alam NE, Islam MS, Ullah H, Molla MT, Shifat SK, Akter S, et al. Evaluation of knowledge, awareness and attitudes towards breast cancer risk factors and early detection among females in Bangladesh: A hospital based cross-sectional study. PLoS One. 2021;16(9):e0257271. doi: 10.1371/journal.pone.0257271 34516589 PMC8437277

[11] Alsaraireh A, Darawad MW. Breast cancer awareness, attitude and practices among female university students: A descriptive study from Jordan. Health Care Women Int. 2018;39(5):571–83. doi: 10.1080/07399332.2017.1368516 28850299

[12] Andegiorgish AK, Kidane EA, Gebrezgi MT. Knowledge, attitude, and practice of breast Cancer among nurses in hospitals in Asmara, Eritrea. BMC Nurs. 2018;17:33. doi: 10.1186/s12912-018-0300-4 30083079 PMC6069844

[13] W H O. WHO supports early detection and control of cervical and breast cancer in Bangladesh. https://www.who.int/bangladesh/news/detail/10-11-2020-who-supports-early-detection-and-control-of-cervical-and-breast-cancer-in-bangladesh. 2020.

[14] Hellquist BN, Duffy SW, Abdsaleh S, Björneld L, Bordás P, Tabár L, et al. Effectiveness of population-based service screening with mammography for women ages 40 to 49 years: evaluation of the Swedish Mammography Screening in Young Women (SCRY) cohort. Cancer. 2011;117(4):714–22. doi: 10.1002/cncr.25650 20882563

[15] Huguley CM Jr, Brown RL, Greenberg RS, Clark WS. Breast self-examination and survival from breast cancer. Cancer. 1988;62(7):1389–96. doi: 10.1002/1097-0142(19881001)62:7<1389::aid-cncr2820620725>3.0.co;2-0 3416278

[16] Anderson BO, Braun S, Lim S, Smith RA, Taplin S, Thomas DB, et al. Early detection of breast cancer in countries with limited resources. Breast J. 2003;9 Suppl 2:S51-9. doi: 10.1046/j.1524-4741.9.s2.4.x 12713497

[17] Ahmed BA. Awareness and practice of breast cancer and breast-self examination among university students in Yemen. Asian Pac J Cancer Prev. 2010;11(1):101–5. 20593937

[18] Siahpush M, Singh GK. Sociodemographic variations in breast cancer screening behavior among Australian women: results from the 1995 National Health Survey. Prev Med. 2002;35(2):174–80. doi: 10.1006/pmed.2002.1063 12200103

[19] Cancer Research UK. Breast cancer incidence (invasive) statistics. Cancer Research UK. https://www.cancerresearchuk.org/health-professional/cancer-statistics/statistics-by-cancer-type/breast-cancer/incidence-invasive

[20] Cancer Research UK. Teenage and young adult (TYA) cancers. https://www.cancerresearchuk.org/about-cancer/childrens-cancer/teenage-young-adult-tya

[21] Ahmadian Samah. A literature review of factors influencing breast cancer screening in Asian countries. ResearchGate. 2012.

[22] Board of Intermediate and Secondary Education, Dhaka: Center-Wise School List For SSC. Zilla: Dhaka Mahanagari, Bangladesh, Dhaka; 2025. https://www.scribd.com/document/842373772/20250224145654992941

[23] Al-Dubai SAR, Ganasegeran K, Alabsi AM, Abdul Manaf MR, Ijaz S, Kassim S. Exploration of barriers to breast-self examination among urban women in Shah Alam, Malaysia: a cross sectional study. Asian Pac J Cancer Prev. 2012;13(4):1627–32. doi: 10.7314/apjcp.2012.13.4.1627 22799379

[24] Boulos DNK, Ghali RR. Awareness of breast cancer among female students at Ain Shams University, Egypt. Global Journal of Health Science. 2013;6:154. doi: 10.5539/gjhs.v6n1p154 24373275 PMC4825266

[25] Karayurt O, Ozmen D, Cetinkaya AC. Awareness of breast cancer risk factors and practice of breast self examination among high school students in Turkey. BMC Public Health. 2008;8:359. doi: 10.1186/1471-2458-8-359 18928520 PMC2587470

[26] Nemenqani DM, Abdelmaqsoud SH, Al-Malki A-HA, Oraija AA, Al-Otaibi EM. Knowledge, attitude and practice of breast self examination and breast cancer among female medical students in Taif, Saudi Arabia. OJPM. 2014;04(02):69–77. doi: 10.4236/ojpm.2014.42011

[27] Ranasinghe HM, Ranasinghe N, Rodrigo C, Seneviratne RDA, Rajapakse S. Awareness of breast cancer among adolescent girls in Colombo, Sri Lanka: a school based study. BMC Public Health. 2013;13:1209. doi: 10.1186/1471-2458-13-1209 24359310 PMC3906911

[28] Sari SYI, Desmona D, Djajakusumah TM. Low Knowledge and Negative Perception about the Risks of Breast Cancer among Female High School Students. amj. 2019;6(3):129–35. doi: 10.15850/amj.v6n3.1675

[29] Karima B, Zineb S, Samir D, Othmani Mohamed B. Awareness of Risk Factors for Breast Cancer among Casablanca Medical Students. Asian Pac J Cancer Care. 2023;8(2):311–7. doi: 10.31557/apjcc.2023.8.2.311-317

[30] Mohebi Z, Heidari Sarvestani M, Moradi Z, Naghizadeh MM. Female high school students’ knowledge and attitude toward breast cancer. BMC Womens Health. 2023;23(1):41. doi: 10.1186/s12905-023-02155-z 36717852 PMC9887865

[31] Shamsuddeen SB, Ansari A, Ali MS. Nutrition knowledge, attitude, practice towards breast cancer prevention among the female students of University of Hail, Saudi Arabia. Med Sci Discov. 2023;10:187–94. doi: 10.36472/msd.v10i3.899

[32] Chen X, Yan Q, Tang Y, Zhu J, Zhang W, Zhang J. Financial toxicity, family resilience and negative emotions among young and middle-aged breast cancer patients: A multicentre cross-sectional study. Breast. 2024;75:103735. doi: 10.1016/j.breast.2024.103735 38640552 PMC11031793

[33] Ngo NTN, Nguyen HT, Nguyen PTL, Vo TTT, Phung TL, Pham AG, et al. Health-related quality of life in breast cancer patients in low-and-middle-income countries in Asia: a systematic review. Front Glob Womens Health. 2023;4:1180383. doi: 10.3389/fgwh.2023.1180383 37389285 PMC10304018

[34] Alwan NAS, Al-Attar WM, Eliessa RA, Madfaie ZA, Tawfeeq FN. Knowledge, attitude and practice regarding breast cancer and breast self-examination among a sample of the educated population in Iraq. East Mediterr Health J. 2012;18(4):337–45. doi: 10.26719/2012.18.4.337 22768695

[35] Sarker R, Islam MS, Moonajilin MS, Rahman M, Gesesew HA, Ward PR. Knowledge of breast cancer and breast self-examination practices and its barriers among university female students in Bangladesh: Findings from a cross-sectional study. PLoS One. 2022;17(6):e0270417. doi: 10.1371/journal.pone.0270417 35763525 PMC9239455

[36] Singh R, Turuk A. A study to assess the knowledge regarding breast cancer and practices of breast self-examination among women in urban area. Int J Community Med Public Health. 2017;4(11):4341. doi: 10.18203/2394-6040.ijcmph20174856

