# Supplementary material for: Knowledge of breast cancer and practice of breast self-examination among adolescent girls in semi-urban area – Savar, Dhaka, Bangladesh: A school-based cross-sectional study
Source: PLoS One. 2026 Jun 11;21(6):e0334375. doi: 10.1371/journal.pone.0334375 (PMC13258002; doi:10.1371/journal.pone.0334375)
Supplement: S1 File — (DOCX) [file pone.0334375.s001.docx]

**Table S1. Distribution of knowledge about symptoms of breast cancer**

| **Variables** | **n** | **(%)** |
| --- | --- | --- |
| **Sagging of breast (Neg Question)** | | |
| No | 113 | (29.4) |
| Yes | 38 | (9.9) |
| Don't know | 233 | (60.7) |
| **Nipple discharge other than breast milk including blood or pus** | | |
| Yes | 96 | (25.0) |
| No | 99 | (25.8) |
| Don't know | 189 | (49.2) |
| **Swelling of part of the breast** | | |
| Yes | 146 | (38.0) |
| No | 91 | (23.7) |
| Don't know | 147 | (38.3) |
| **Shrinking of breast skin** | | |
| Yes | 92 | (24.0) |
| No | 93 | (24.2) |
| Don't know | 199 | (51.8) |
| **Changes in breast shape and size** | | |
| Yes | 83 | (21.6) |
| No | 89 | (23.2) |
| Don't know | 212 | (55.2) |
| **Color change of breast including redness or flaky skin** | | |
| Yes | 50 | (13.0) |
| No | 100 | (26.0) |
| Don't know | 234 | (60.9) |
| **New lump in the breast or armpit** | | |
| Yes | 75 | (19.5) |
| No | 110 | (28.6) |
| Don't know | 199 | (51.8) |

***Overall right answer is 24.36%***

**Table S2. Distribution of knowledges about risk factors of breast cancer.**

| **Variables** | **n** | **(%)** |
| --- | --- | --- |
| **Not feeding breast milk to children** | | |
| Yes | 50 | (13.0) |
| No | 83 | (21.6) |
| Don't know | 251 | (65.4) |
| **Not being physically active** | | |
| Yes | 51 | (13.3) |
| No | 66 | (17.2) |
| Don't know | 267 | (69.5) |
| **Previous treatment with hormones or radiation** | | |
| Yes | 70 | (18.2) |
| No | 48 | (12.5) |
| Don't know | 266 | (69.3) |
| **Food habit** | | |
| Yes | 88 | (22.4) |
| No | 67 | (17.4) |
| Don't know | 231 | (60.2) |
| **Cyst in Breast** |  |  |
| Yes | 114 | (29.7) |
| No | 35 | (9.1) |
| Don't know | 235 | (61.2) |
| **Genetic reasons/Family history** | | |
| Yes | 106 | (27.6) |
| No | 39 | (10.2) |
| Don't know | 239 | (62.2) |
| **Alcohol consumption** | | |
| Yes | 89 | (23.2) |
| No | 56 | (14.6) |
| Don't know | 239 | (62.2) |
| **Ageing/Getting older** | | |
| Yes | 45 | (11.7) |
| No | 77 | (20.1) |
| Don't know | 262 | (68.2) |
| **Consuming birth control pill regularly** | | |
| Yes | 35 | (9.1) |
| No | 49 | (12.8) |
| Don't know | 300 | (78.1) |
| **Obesity** |  |  |
| Yes | 41 | (10.7) |
| No | 52 | (13.5) |
| Don't know | 291 | (75.8) |

***Overall right answer is 17.94%***

**Table S3. Distribution of knowledges about treatment of breast cancer.**

| Variables | n | (%) |  |
| --- | --- | --- | --- |
| **Breast cancer is curable if detected at early stage** | | |  |
| Yes | 161 | (41.9) |  |
| No | 28 | (6.8) |  |
| Don't know | 197 | (51.3) |  |
| **Chemotherapy is an effective treatment of breast cancer** | | |  |
| Yes | 159 | (41.4) |  |
| No | 18 | (4.7) |  |
| Don't know | 207 | (53.9) |  |
| **Surgery is an effective treatment of breast cancer** | | |  |
| Yes | 115 | (29.9) |  |
| No | 25 | (6.5) |  |
| Don't know | 244 | (63.5) |  |
| **Radiotherapy is an effective treatment of breast cancer** |  |  |  |
| Yes | 45 | (11.7) |  |
| No | 33 | (8.6) |  |
| Don't know | 306 | (79.7) |  |
| **Hormonal therapy is an effective treatment of breast cancer** | | |  |
| Yes | 91 | (23.7) |  |
| No | 21 | (5.5) |  |
| Don't know | 272 | (70.8) |  |
| **Curable by Alternative medicines (Neg Question)** | | |  |
| No | 47 | (12.2) |  |
| Yes | | 62 | (16.1) |
| Don't know | 275 | (71.6) |  |
| **Curable by herbal treatment (Neg Question)** | | |  |
| No | 64 | (16.7) |  |
| Yes | 45 | (11.7) |  |
| Don't know | 275 | (71.6) |  |

***Overall right answer is 27.47%***

**Table S4. Distribution of knowledges about prevention of breast cancer.**

| **Variables** | **n** | **(%)** |
| --- | --- | --- |
| **Breast cancer is 100% preventable** | | |
| Yes | 73 | (19.0) |
| No | 68 | (17.7) |
| Don't know | 243 | (63.3) |
| **Feeding breastmilk to child regularly** | | |
| Yes | 85 | (22.1) |
| No | 65 | (16.9) |
| Don't know | 234 | (60.9) |
| **Not wearing underwear all the time** |  |  |
| Yes | 74 | (19.3) |
| No | 34 | (8.9) |
| Don't know | 276 | (71.9) |
| **Early detection and seeking medical assistance if any symptoms is found** | | |
| Yes | 152 | (39.6) |
| No | 12 | (3.1) |
| Don't know | 220 | (57.3) |
| **Maintaining ideal body weight** | | |
| Yes | 218 | (56.8) |
| No | 16 | (4.2) |
| Don't know | 150 | (39.1) |
| **Being physically active** | | |
| Yes | 171 | (44.5) |
| No | 7 | (1.8) |
| Don't know | 206 | (53.6) |
| **Vaccine (Neg Question)** | | |
| No | 14 | (3.6) |
| Yes | 164 | (42.7) |
| Don't know | 168 | (43.8) |
| **Food habit** |  |  |
| Yes | 202 | (52.6) |
| No | 14 | (3.6) |
| Don't know | 168 | (43.8) |

***Overall right answer is 32.19%***

**Table S5. Distribution of knowledge about screening of breast cancer.**

| **Variables** | **n** | **(%)** |
| --- | --- | --- |
| **Clinical examination is a type of screening** | | |
| Yes | 119 | (31.0) |
| No | 13 | (3.4) |
| Don't know | 252 | (65.6) |
| **Mammography is a type of screening** | | |
| Yes | 35 | (9.1) |
| No | 26 | (6.8) |
| Don't know | 323 | (84.1) |
| **Breast self-examination is a type of screening** | | |
| Yes | 58 | (15.1) |
| No | 10 | (2.6) |
| Don't know | 316 | (82.3) |
| **Ultrasound is a type of screening** | | |
| Yes | 57 | (14.8) |
| No | 23 | (6.0) |
| Don't know | 304 | (79.2) |

***Overall right answer is 17.51%***

**Table S6. Distribution of knowledge about process of breast self-examination.**

| **Variables** | **n** | **(%)** |
| --- | --- | --- |
| **Inspecting breast visually Infront of a mirror to look for any changes like size, shape, color, unusual discharge or nipple inversion** | | |
| Yes | 91 | (23.7) |
| No | 35 | (9.1) |
| Don't know | 258 | (67.2) |
| **Inspecting breast and look for changes Infront of mirror by holding arms at sides, by arms over head, by hands on hips and tighten chest muscle, and by bending forward with hands on hips** | | |
| Yes | 74 | (19.3) |
| No | 25 | (6.5) |
| Don't know | 285 | (74.2) |
| **Inspecting breast by lying down on back with pillow under shoulder and use pads of three middle fingers to give pressure in circle, up and down pattern for each breast** | | |
| Yes | 75 | (19.5) |
| No | 30 | (7.8) |
| Don't know | 279 | (72.7) |
| **Feel for changes in armpits by fingers in up down vertical** | | |
| Yes | 58 | (15.1) |
| No | 33 | (8.6) |
| Don't know | 293 | (76.3) |
| **Inspecting breasts while bathing with soap** | | |
| Yes | 58 | (15.1) |
| No | 47 | (12.2) |
| Don't know | 279 | (72.7) |

***Overall right answer is 18.54%***
